# Evaluating *Lymantria dispar* Mating Disruption in Southeastern Europe via Male Flight Activity and Egg Clusters

**DOI:** 10.3390/insects17050470

**Published:** 2026-05-02

**Authors:** Maria C. Boukouvala, Anna Skourti, Demeter Lorentha S. Gidari, Constantin S. Filintas, Nickolas G. Kavallieratos

**Affiliations:** Laboratory of Agricultural Zoology and Entomology, Faculty of Crop Science, Agricultural University of Athens, 75 Iera Odos Str., 11855 Athens, Greece; mbouk@aua.gr (M.C.B.); annaskourti@aua.gr (A.S.); dlgidari@aua.gr (D.L.S.G.); kfilintas@aua.gr (C.S.F.)

**Keywords:** spongy moth, forest pest, Europe, management, reproduction inhibition, eggs

## Abstract

The spongy moth, *Lymantria dispar* (L.) (Lepidoptera: Erebidae), is one of the most destructive defoliators, primarily of oak forest ecosystems, causing severe ecological and economic damages. The present study evaluated the effectiveness of the mating disruption method against *L. dispar* populations in an oak-dominated forest in Greece. Field experiments were conducted for three consecutive years (2022–2024) in two forest areas: a treated area, where a flowable pheromone gel was applied, and an untreated control area. The *L. dispar* male flight activity was monitored using pheromone-baited traps. Egg cluster counts were assessed during winter to evaluate reproductive success. The obtained results revealed a significant reduction in male trap captures in the treated area, following the mating disruption application in 2023 and 2024, compared to the control area. Additionally, the egg cluster counts decreased significantly in the mating disruption area, reaching reductions of 94.8% and 99.2% during the winters of 2023–2024 and 2024–2025, respectively, compared to the winter of 2022–2023 (before the mating disruption application). These results demonstrate that mating disruption, an environmentally friendly strategy for sustainable forest pest management, can effectively reduce *L. dispar* populations under Mediterranean environmental conditions.

## Simple Summary

The present study evaluated the effectiveness of the mating disruption method against *Lymantria dispar* in Greece. The experiment was conducted over three years and compared the treated area with an untreated control. Male flight activity was monitored using pheromone-baited traps, and reproduction success was assessed by counting egg clusters during winter surveys. The results demonstrated a significant reduction in the *L. dispar* male flight activity and the egg cluster counts in the treated area, compared to the control. These findings indicate that mating disruption can be an effective and sustainable approach against *L. dispar* populations in Mediterranean oak forests.

## 1. Introduction

Forest ecosystems offer vital ecological, economic, and social services at local and global levels [1,2,3], playing a critical role in the production of timber and other natural products, biodiversity preservation, climate regulation, and carbon sequestration [4,5,6,7]. Nonetheless, a variety of biotic (e.g., pests, pathogens) and abiotic (e.g., drought, high temperature) stressors pose a constant threat to forest ecosystems [8,9,10]. It is well known that forests face serious losses due to insect herbivore species [9,11,12] that have the potential to reach extensive outbreaks [12,13]. Large-scale defoliation, increased vulnerability to diseases and secondary pests, and eventually death of trees are all consequences of forest insect pests. These disturbances change the plant species composition and structure of forests, weaken ecosystem stability, and cause significant financial losses to the forestry industry (e.g., high timber loss, reduced tourism revenues) [13,14,15].

One of the most destructive forest defoliators worldwide is the spongy moth, *Lymantria dispar* (L.) (Lepidoptera: Erebidae) [16]. Larvae of *L. dispar* infest more than 300 tree species’ foliage during spring [17,18,19,20]. Although oaks (*Quercus* spp.) are the preferred hosts, a variety of tree species, including birch (*Betula* spp.), beech (*Fagus* spp.), and poplar (*Populus* spp.), are also susceptible [21,22,23]. There are four distinct developmental phases in the life cycle of the univoltine *L. dispar*: egg, larva, pupa, and adult. In late summer, females lay clutches of eggs on rocks, tree bark, and artificial surfaces (e.g., roofs, walls, cars), protecting the species to overwinter in the egg stage for 8–9 months [17,21,24,25,26]. Egg clusters, which are coated with hairs from the female’s abdomen and contain several hundred eggs, offer defense from environmental conditions and predators [19]. Early instar larvae can spread through a behavior known as ballooning, by producing silk threads that enable them to be blown by the wind to other host trees [27,28,29]. Larvae pupate in protected areas such as branches or cracks in bark after completing their development. Adults emerge in the middle to late summer, exhibiting extensive sexual dimorphism [30,31,32]. Brown, fully winged, and capable of sustained flight, males are able to follow sex pheromone plumes to detect females. Females, on the other hand, are unable to fly (the European subspecies) and they use pheromone emission to attract potential mates, even within two hours of pupal eclosion. The exposure of the abdominal final segments and a rhythmic pumping motion—an action known as “calling”—are associated with the production of pheromones. Instead of being released constantly throughout the day, pheromones are released in spurts of calling activity. Males can be attracted to pheromone from long distances. They follow the odor trail in the air with an erratic flight pattern, tracking the progressively strong gradient of the odor. Visual cues are also used to spot the female, probably due to the contrast between the light-colored female and the dark trunk of the tree. During courtship, a male approaches a female, and the copulation occurs when the female lifts her wings to allow mating. Although copulation can take up to an hour, the spermatophore or sperm packet typically passes during the first 10 minutes [19,30,33,34].

To reduce *L. dispar* populations and decrease their effects, various management strategies have been developed, including synthetic insecticides, predators, parasitoids, and pathogens [26,32,35,36,37,38]. However, control measures, including synthetic insecticides, face disadvantages, such as regulations, effects on non-target species, and development of resistance [39,40,41]. Mating disruption (MD), i.e., the release of artificial female pheromones in the area to camouflage and compete with pheromone produced by the calling females, drastically reduces mate detection and eventually mating [42]; this has long been considered an effective non-chemical control method, particularly for Lepidoptera [43,44,45,46,47]. This method can delay mating, thereby interrupting fertility and egg fertilization [48,49]. Especially for *L. dispar*, the MD method has demonstrated strong efficacy against low-density populations, particularly in the USA [50,51,52,53,54,55,56]. In addition to the success of the method in the USA, MD is a step toward more ecologically sustainable pest management frameworks. This is especially important for the maintenance of beneficial species’ populations (e.g., pollinators, predators, and parasitoids) [57], key elements of the forest biodiversity [10].

In Europe, the knowledge about the MD method against *L. dispar* is limited, particularly in Western and Central Europe [50,58]. In Southeastern Europe, the MD method has never been evaluated to control *L. dispar*. Particularly in Greece, until now, the studies on the control methods against *L. dispar* have involved laboratory and semi-field experiments using synthetic or natural insecticides [59,60,61]. However, there is a lack of large-scale management in Greek forests. As the biology and the flight behavior vary with different climates and ecosystems [62,63,64], it is important to evaluate the effectiveness of the MD method under Mediterranean environmental conditions. Thus, the present study applied this method against *L. dispar* in Greece. The efficacy of the method was assessed by monitoring the male flight activity in the tested areas [44]. Additionally, the study assessed, for the first time in Europe, the density of the egg clusters, a crucial indicator for the evaluation of the success of the MD method [65,66].

## 2. Materials and Methods

### 2.1. Experimental Areas

**Figure 1:**
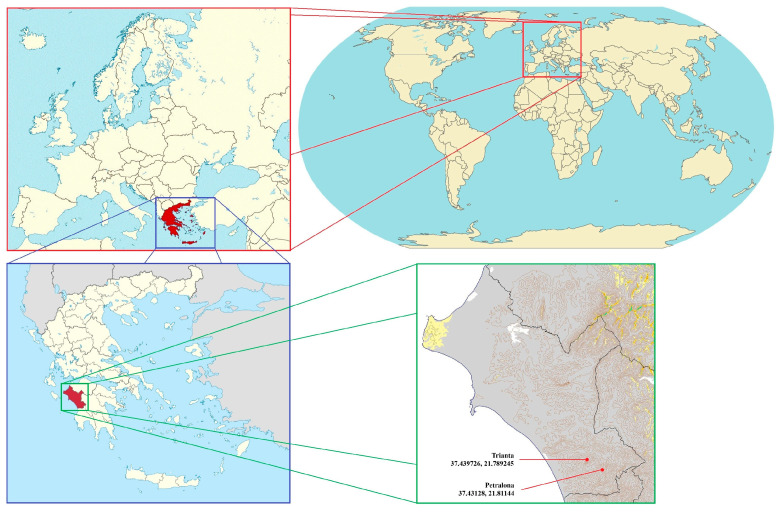
Map of the experimental areas in Greece: Trianta and Petralona, in the Prefecture of Ilia, Peloponnese.

The application of the MD method was carried out in Trianta, located in the Prefecture of Ilia (Peloponnese, Greece) [37.439726, 21.789245] (Figure 1). This area covers 9.136880 hectares (ha). Another area in the same prefecture, namely Petralona, was used as a control. Petralona covers an area of 10.933670 ha [37.43128, 21.81144] (Figure 1). In both areas, vegetation mainly consists of trees of the *Quercus* genus, which are 1–6 m tall and have a density of 100 trees per ha. Trianta and Petralona are forested areas. No control methods were used before and during the experimentation. Additionally, both areas were selected because of heavy *L. dispar* infestation [63,64], based on the monitoring of larvae, adult male population, and count of the egg clusters, as well as for easy access via provincial roads.

### 2.2. Experimental Setup

**Figure 2:**
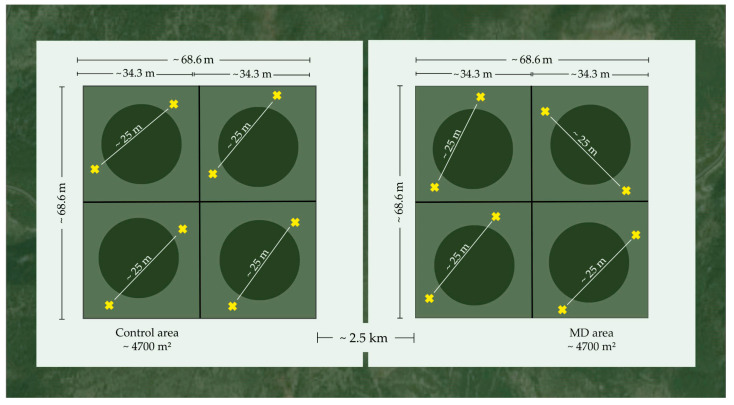
Experimental setup. The dark green circles (Ø 20 m) represent the plots used to record *Lymantria dispar* egg clusters. The yellow crosses represent the pheromone traps. The green squares represent the experimental blocks.

A plot of approximately 4700 m^2^ was selected from the Trianta area (MD) and a similar sized plot from the Petralona area (control). Both plots were divided into 4 blocks [46], as shown in Figure 2, and were used throughout the experimental period. The distance between the two plots was approximately 2.5 km (Figure 2). Within each block, 2 eGYMER 1 traps [63] were placed (i.e., 8 traps for each area) for the monitoring of flight performance of adult males pre- and post-MD application. Each trap was baited with disparlure (Novagrica, Attica, Greece), a flowable paste that contains 0.001 g of Z 7,8 epoxy-2-methyloctadecane, a component of the female sex pheromone [63]. The distance between traps within the same block was about 25 m (Figure 2).

**Figure 3:**
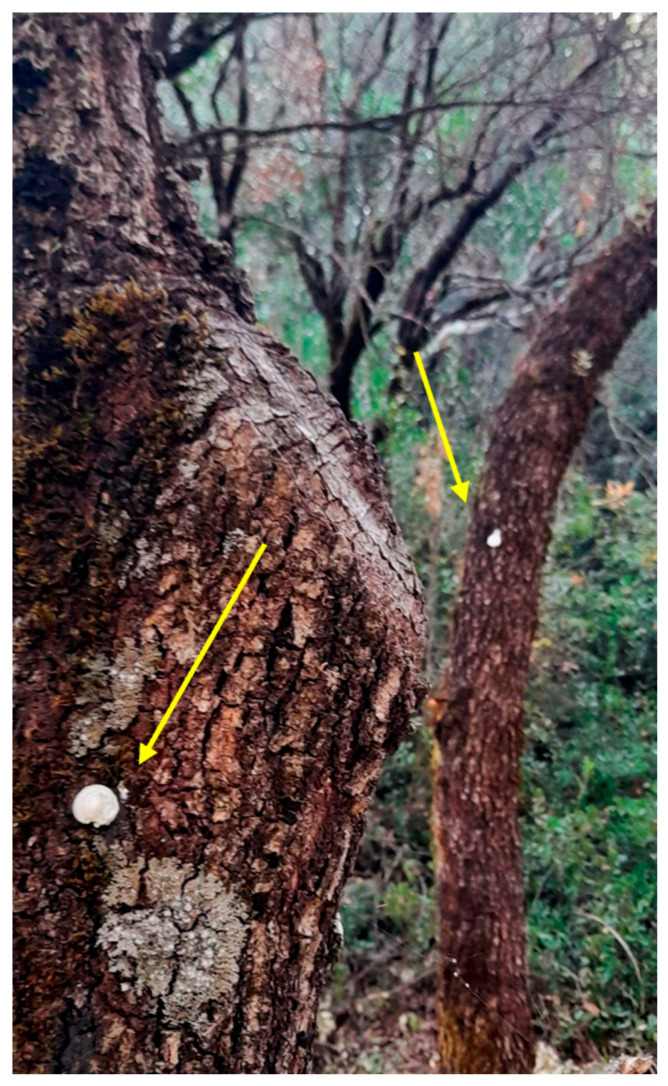
The drops of the MD gel placed on the trunks of *Quercus* trees.

The traps were checked every 3 or 5 days during 2022 and once a week during 2023 and 2024 until the termination of the flight activity, recording and removing trapped males. The Trianta (MD) area was treated with MD gel (Novagrica, Attica, Greece), which is a flowable formulation containing 3.3% (*w*/*w*) of the *L. dispar* sex pheromone, namely cis-7,8-epoxy-2-methyloctadecene [50]. By pressing the package, οne drop of gel (~3 g) (Figure 3) was applied on each tree trunk (at 1.5 m height) within the MD area, using about 470 g of gel for each experimental year. As the gel formulation contains 3.3% active ingredient (a.i.) and the experimental area covers 4700 m^2^ (~½ hectare (ha)), the quantity of the a.i. per ha is 31 g (470 g × 3.3/100 = 15.5 g per~½ ha = 31 g per~ha). In 2023 and 2024, the MD gel was applied on the day of the first captures of *L. dispar* male adults in the traps, i.e., in 23 June 2023 and 29 May 2024, while in 2022, the application of the MD gel was carried out toward the end of the flight activity (i.e., 31 July 2022), because gel became available late in the *L. dispar* flight season.

**Figure 4:**
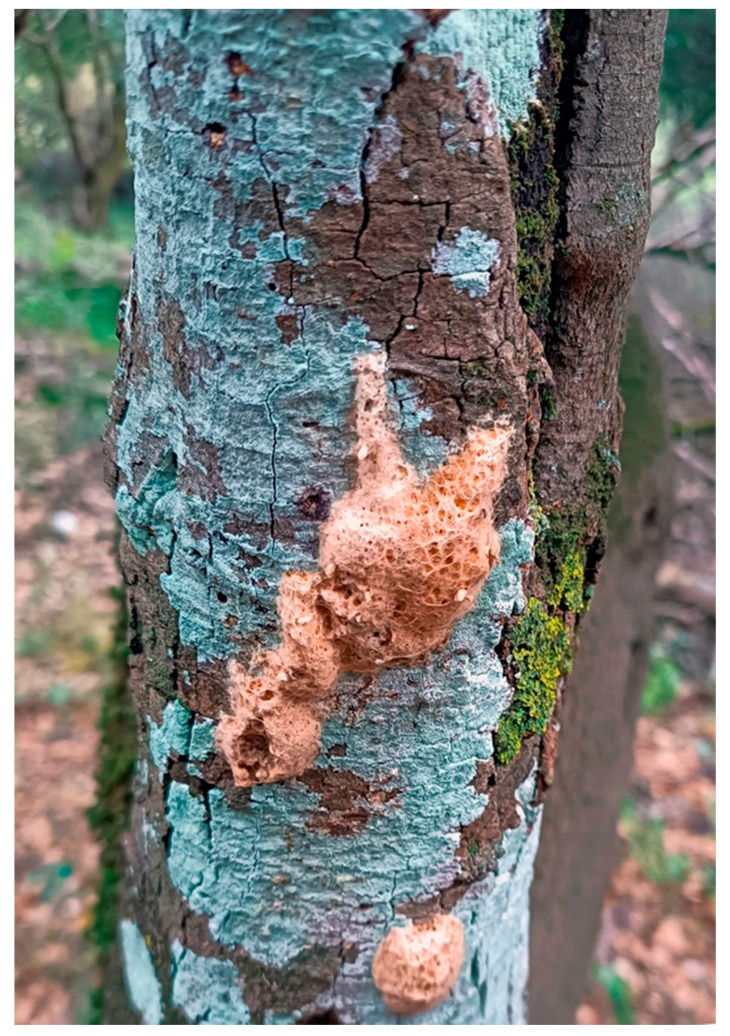
Egg clusters of *Lymantria dispar* on the trunk of a *Quercus* tree.

In addition, in the winter of 2022–2023, 2023–2024, and 2024–2025, the number of egg clusters (Figure 4) was counted in both MD and control areas, according to the method of Liebhold et al. [67] with some modifications. Specifically, within each block, we created a circle with a radius of ~10 m (Figure 2), where we recorded the egg clusters in this area each winter. Thus, 4 circles were created in the MD area and 4 in the control area. The proportion of the reduction in the egg clusters in each area was then calculated using the following formula: (%) reduction in egg clusters = (number of egg clusters before MD gel application—number of egg clusters in the first or second year after MD application)/ number of egg clusters before MD application × 100%. Distinguishing new egg clusters from the old ones can be done visually (new egg clusters are darker in color and less rough in appearance than old egg masses) and by touch (new egg clusters are fuller and harder than old egg masses, which are spongier and softer) [67].

### 2.3. Statistical Analysis

The data on the captured males in the control and MD areas were modified with log (x + 1) prior to analysis to achieve normal variances and standardize means [68,69]. Within each trap check day, a two-tailed *t*-test was used to compare the mean values between captures from the control and MD areas, at the 0.05 significance level [70]. Regarding the counts of egg masses, a Generalized Linear Model (GLM) with a Poisson distribution and a log link function was used for the analysis [71,72]. The main factors included treatment, experimental year, and their interaction (i.e., treatment × experimental year). Additionally, each year was analyzed separately using Poisson GLM analyses to determine statistically significant differences between the control and MD areas. The differences between values were considered significant at the probability level *p* ≤ 0.05. The data analysis of captured males and egg clusters was performed using JMP 16.2 software [73].

## 3. Results

### 3.1. Trapped Lymantria dispar Males

#### 3.1.1. Trapped *Lymantria dispar* Males in 2022

**Figure 5:**
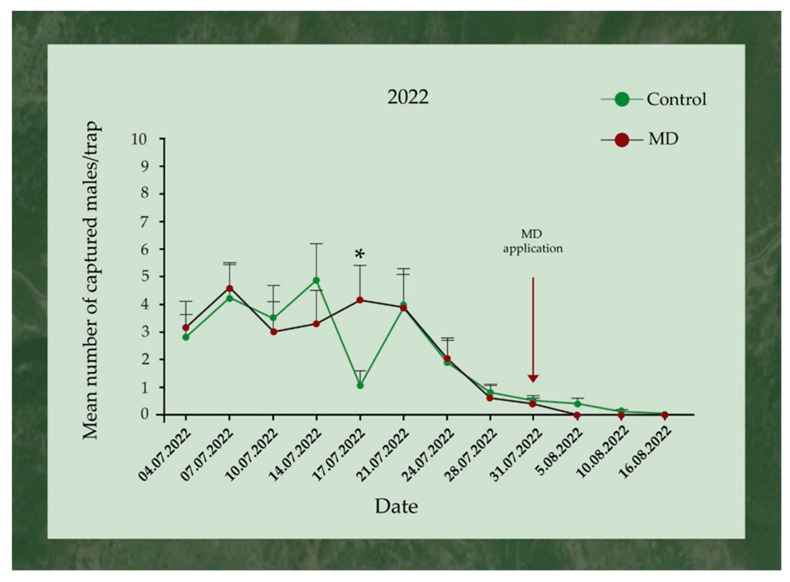
Mean (+SE) number of *Lymantria dispar* adult males trapped in pheromone traps (eGYMER 1) in Trianta (MD area) and Petralona (control area) during 2022. Within each trap check date, significant differences are indicated by asterisks (two-tailed *t*-test at *p* = 0.05, df = 14).

The first captures of *L. dispar* males, in both control and MD areas (22 and 25 trapped males, respectively), were observed on 4th of July, while the last ones were observed on 5th August for the MD area and on 16th August for the control. Before the application of the gel, the mean number of *L. dispar* males trapped in the control area peaked on 14th July, while in the MD area they peaked on the 7th of the same month. The application of the gel marked the end of captures in the MD area, while in the control area, 3 and 1 *L. dispar* males were trapped 5 and 10 days after, respectively. The mean numbers of *L. dispar* males trapped in the control and the MD areas for each inspection day during 2022 are presented in Figure 5.

#### 3.1.2. Trapped *Lymantria dispar* Males in 2023

**Figure 6:**
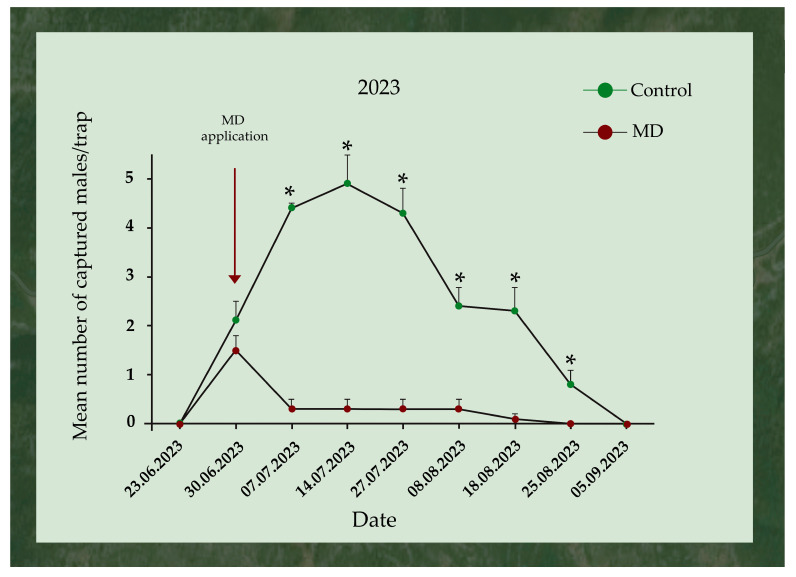
Mean (+SEM) number of *Lymantria dispar* adult males trapped in pheromone traps (eGYMER 1) in Trianta (MD area) and Petralona (control area) during 2023. Within each trap check date, significant differences are indicated by asterisks (two-tailed *t*-test at *p* = 0.05, df = 14).

During 2023, the application of the gel took place when the first *L. dispar* males were captured in the traps. On the first day of the application, the mean number of captured *L. dispar* males per trap between the control and the MD area did not differ significantly (17 and 12 trapped males, respectively). In contrast, all the hereafter inspections (7th, 14th, and 27th July and 8th, 18th, and 25th August) revealed significant differences in the mean number of captured *L. dispar* males per trap between the two tested areas (control and MD). The *L. dispar* male captures for the MD area ended on 25th August, while they ended on 5th September for the control area (Figure 6).

#### 3.1.3. Trapped *Lymantria dispar* Males in 2024

**Figure 7:**
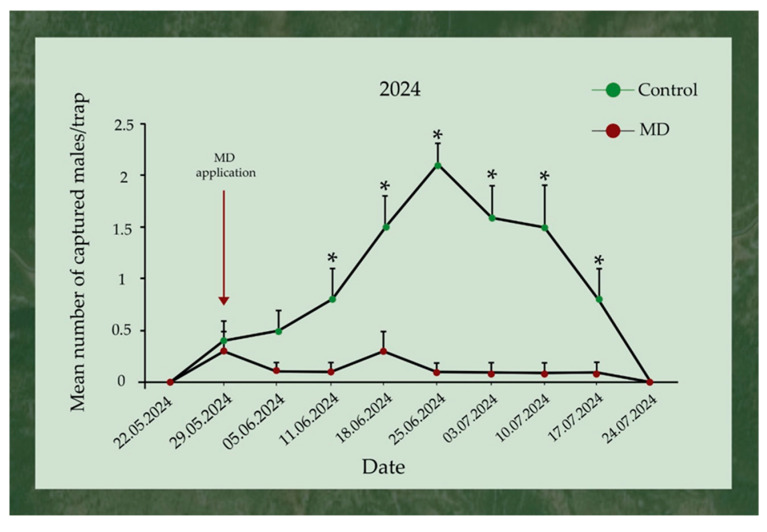
Mean (+SEM) number of *Lymantria dispar* adult males trapped in pheromone traps (eGYMER 1) in Trianta (MD area) and Petralona (control area) during 2024. Within each trap check date, significant differences are indicated by asterisks (two-tailed *t*-test at *p* = 0.05, df = 14).

The mean number of captured *L. dispar* males per trap between the two tested areas (control and MD) started to significantly differ two weeks after the application (11th June), until 24th July. Specifically, on 11th, 18th, 25th June and 3rd, 10th, and 17th July, the trapped males at the control vs. the MD areas were 6 vs. 1, 12 vs. 2, 17 vs. 1, 13 vs. 1, 12 vs. 1, and 6 vs. 1, respectively. The captures stopped on 24th July for both control and MD areas (Figure 7).

### 3.2. Egg Clusters

**Figure 8:**
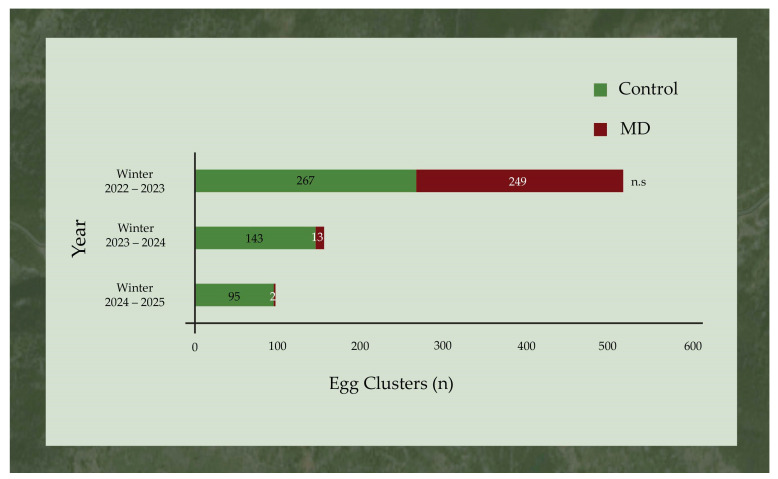
Total number of *Lymantria dispar* egg clusters per experimental year in Trianta (MD area) and Petralona (control area). Within each year, asterisks indicate significant differences between the two areas; n.s. indicates no significant differences between the two areas (Generalized Linear Model, Poisson distribution, *p* ≤ 0.05).

In 2023–2024, a 94.8% reduction in egg clusters was recorded in the MD area after the application of the gel, whereas in 2024–2025, this proportion increased to 99.2%. A reduction in the counts of egg clusters was also recorded in the control area. More specifically, a 46.4% and a 64.4% reduction in egg clusters were observed during the winter of 2023–2024 and winter of 2024–2025, respectively, compared to 2022. The Poisson GLM analysis showed a statistically significant main effect of treatment (*χ*^2^ = 11.7, df = 1, *p* = 0.001) and experimental year (*χ*^2^ = 24.2, df = 2, *p* < 0.001), as well as a significant treatment x experimental year interaction (*χ*^2^ = 9.4, df = 2, *p* = 0.009). Furthermore, individual analysis by year revealed that there was no significant difference between the number of egg clusters found in the control (i.e., 267 egg clusters) and the MD area (249 egg clusters) in the winter of 2022–2023 (*χ*^2^ = 0.8, df = 1, *p* = 0.37) (Figure 8). The application of the gel in the MD area in 2023 resulted in a significantly lower number of egg clusters (13 egg clusters) in comparison to the control area (143 egg clusters) (*χ*^2^ = 8.9, df = 1, *p* = 0.003). Similarly, one year later and after the gel treatment, the number of egg clusters in the control area was significantly higher compared to the MD area (*χ*^2^ = 4.6, df = 1, *p* = 0.032).

## 4. Discussion

By monitoring both the male flight activity and the egg cluster density over a three-year period the present study demonstrated that the flowable pheromone gel achieved significant reduction in *L. dispar* population development in an oak-dominated forest ecosystem in Southeastern Europe and particularly Greece. Monitoring the *L. dispar* male captures revealed a clear disruption of male orientation to pheromone-baited traps in the MD area after the gel application in 2023 and 2024. Similarly, the MD method resulted in the disruption of the flight, mating behavior, and reproduction success of other noxious lepidopterans of forest and agricultural importance, such as *Thaumetopoea pityocampa* (Denis and Schiffermüller) (Lepidoptera: Thaumetopoeidae), *Helicoverpa armigera* (Hübner) (Lepidoptera: Noctuidae), *Apomyelois ceratoniae* (Zeller) (Lepidoptera: Pyralidae), and *Tuta absoluta* (Meyrick) (Lepidoptera: Gelechiidae) [46,74,75,76]. Similar efficacy was observed in previous research conducted primarily in North America, the United Kingdom, and Slovenia, where pheromone-based disruption has been successfully incorporated into integrated management programs for *L. dispar* [50,51,56,58,65,66,77,78]. For instance, studies conducted within the USDA Slow-the-Spread program, using dispensers of controlled pheromone release, reported reductions in more than 96% in male trap catches and more than 98% in mating success in treated plots compared with untreated controls, at the application rates of 15 and 37.5 g a.i./ha, in the Appomattox-Buckingham and Cumberland Counties, Virginia, USA [79]. Similarly, ground applications of pheromone formulations in the Goshen Wildlife Management Area (GWMA) located in Rockbridge and Augusta Counties, Virginia, USA, reduced male trap catches by more than 90% at the doses of 49.4 and 123.6 g a.i./ha [54]. In the current study, the application rate of 31 g a.i./ha was used, an upper average rate compared to those of Thorpe et al. [79] (15 and 37.5 g a.i./ha) and lower than the doses of Onufrieva et al. [54] (49.4 and 123.6 g a.i./ha). The present and the previous results of the MD against *L. dispar* enhance the applicability of the method in a broad geographical spectrum under variable climatic conditions, in accordance with previous research that evaluated the efficacy of the method against the tested species under various climates [80].

In addition to the male capture suppression, the egg cluster indicator highlighted the efficacy of the method. During the winters of 2023 and 2024, when the MD application was properly timed, the number of egg clusters significantly decreased in the MD area, compared to the control. These findings confirm that MD method does confuse the *L. dispar* male flight and effectively reduce the offspring production of this species [53]. A gradual reduction in egg clusters was also observed in the control areas over the three-year experimental period. Presumably, the presence of pheromones in the MD area affected the mating of *L. dispar* in the nearby control area, given that *L. dispar* pheromones are considered to have an attraction range of 300 m to 15 km [81]. However, it should be noted that a different study proposed that the pheromone activity is limited to ~250 m in most areas and up to 600 m in the mountainous terrain due to the wind tunnel effect [82]. Previous research on mating disruption has shown similar effects, with decreased population indicators (nests) of *T. pityocampa* outside the treated blocks (control area) in Southern Greece and Italy [46]. Since this phenomenon seems to promote mating success reduction in treated and nearby untreated areas, it highlights the importance of MD application on an annual basis. By partially protecting nearby forest stands, this effect may offer an extra benefit from a cost-effective management perspective. Thus, further research needs to be done to verify the assumption of the pheromone effect in the control areas.

The timing of the gel application appeared to play the most critical role in terms of the effectiveness of the method. In 2022, due to the late MD application, only a limited effect on male captures was observed, and the treatment was not expected to influence egg cluster densities recorded during the following winter. In contrast, in 2023 and 2024, when MD was applied at the beginning of the male flight activity, the method resulted in significantly reduced male captures and reduced egg cluster counts. This observation emphasizes the importance of the precise MD application time [44], before or at the onset of the male flight activity.

An additional important aspect of MD is the compatibility of the method with environmentally sustainable management strategies [57,83]. In contrast to broad-spectrum insecticides, pheromone-based methods target a single species and have minimal impact on non-target organisms such as beneficial insects [84,85]. In forest environments, where complex ecological interactions are crucial to preserving biodiversity and ecosystem stability, this selectivity is advantageous [1,2,10]. Therefore, the development of more ecologically friendly pest management systems that reduce chemical inputs while maintaining ecosystem integrity may benefit from the effective MD application for *L. dispar* in Mediterranean forests.

## 5. Conclusions

Overall, the present results indicate that MD is an effective strategy for the management of *L. dispar* populations in Mediterranean oak forests. An interesting direction for future research is the evaluation of the MD over larger spatial scales, that may provide a wider understanding of how environmental factors, forest composition, and population density influence the effectiveness of the method. Additionally, a longer-term application of MD and the monitoring of the effectiveness indicators could provide further insights into the population suppression and the spectrum of the side effects in the untreated areas. The collection and evaluation of such results would contribute to optimizing application strategies and support the integration of MD into management programs for *L. dispar*.

## Data Availability

The original contributions presented in this study are included in the article. Further inquiries can be directed to the corresponding author.
